# A novel belief rule base expert system with interval-valued references

**DOI:** 10.1038/s41598-022-10636-8

**Published:** 2022-04-26

**Authors:** Chao Sun, Ruohan Yang, Wei He, Hailong Zhu

**Affiliations:** 1 Harbin Normal University, Harbin, 150025 China; 2 Northwestern Polytechnical University, Xi’an, 710072 China; 3 Rocket Force University of Engineering, Xi’an, 710025 China

**Keywords:** Computer science, Information technology

## Abstract

As an essential parameter in the belief rule base (BRB), referential values refer to evaluation criteria for describing attributes using quantitative data or linguistic terms, the rationality and preciseness of which are important to the modeling accuracy. At present, the studies on referential values of BRB are mainly related to single-valued data. However, due to the inherent uncertainty, ambiguity, and vagueness of expert knowledge, the single-valued references provided by experts cannot represent qualitative information adequately. In this paper, a novel BRB with interval-valued references (BRB-IR) is proposed, in which qualitative knowledge and quantitative data can be integrated to construct models. First, the interval-valued referential values provided by experts are optimized by a nonlinear optimization algorithm to obtain the optimal referential values. Furthermore, other model parameters are optimized by the projection covariance matrix adaptation evolutionary strategy (P-CMA-ES) algorithm. Finally, a case study for pipeline leak detection is constructed to verify the model's effectiveness, and the results show that the proposed BRB-IR is more effective and characterizes expert knowledge better than the classical BRB using single-valued references.

## Introduction

Expert systems are computer systems with decision-making capabilities that use expert knowledge to infer and obtain interpretable results^1^. As an important expert system, BRB is derived from the Dempster rule and traditional IF–THEN rules^2^. By adding the belief structure to the fuzzy logic framework, BRB addresses various uncertainties^3^. In the construction of BRB-based methods, qualitative information and quantitative data can both be considered, and the modeling process and output are interpretable. Thus it has been applied in many fields, such as risk assessment^4,5^, medicine decision^6,7^, safety assessment^8^, and production planning^9^.

Recently, many scholars have improved the modeling ability and performance of BRB. To solve the combinatorial explosion problem, Cao et al. proposed a single-attribute BRB (ABRB) and proved that its modeling ability is similar to that of conventional BRBs^10^. Yang et al. argued that ABRB ignores the inherent weakness of the single attribute selection method, so multiple attribute selection methods are used to generate multiple BRBs, and then a new cautious conjunctive rule is used to combine the outputs of the BRBs to obtain the modeling result^11^. For a single generated BRB, Hu et al. proposed a distributed BRB modeling and inference method, which divides the BRB into multi-independent subsets and activates one suitable subset when receiving the input data to reduce the modeling complexity and inference cost^12^. To generate initial parameters for large-scale BRB, Zhang et al. picked up the standard rules and given the corresponding rule parameters, and then used the cloud model to automatically generate the parameters of the remaining rules^13^. Guan et al. proposed a momentum stochastic gradient descent BRB to solve the rule zero activation problem, which applied the Gaussian function to calculate rule activation weight. Meanwhile, by discarding the rule weight and introducing a distance-sensitive parameter to each attribute, the calculation of rule activation weight is simplified^14^. To downsize the BRB and enhance its modeling performance, Gao et al. proposed a greedy-based BRB model, which constructs the initial BRB by selecting some rules from candidate rules, and then performs parameter learning to add the optimal rules to the initial BRB, thus removing noise and redundant rules^15^. Extended BRB is a variant of traditional BRB. To enhance its generalization ability, Chen et al. used the *K*-means tree to improve the efficiency of rule search and the random *K*-means clustering forest to improve its modeling accuracy and stability^16^. To make BRB more general, Zhu et al. proposed an interval-valued belief rule inference methodology based on evidential reasoning (IRIMER), which introduced the interval belief degree caused by interval data into BRB^17^. These methods extend the modeling capabilities of BRB, but none of them carry out further studies on attribute references.

In the original BRB, the references of antecedents are single-valued data given by experts and not modified in the whole modeling process, which is the referential values processing method that has been widely adopted. Attributes and their references determine the size of the rule base and affect the accuracy of modeling, so references need to be determined properly and accurately. To optimize the references, Chang et al. studied a parameter learning method for BRB, which identifies key citations for each attribute, thereby constructing a rule-reduced BRB^18^, but it ignores expert knowledge in optimization. To avoid generating a large-scale BRB for a complex problem, Fu et al. utilized the decision tree to generate rules and introduced interval references into BRB, optimized references along with attribute weights, rule weights, and belief degrees, and finally obtained the optimized references^19^. However, different from other parameters, reference represents the objective criteria and should not be treated the same as other parameters. For example, Feng et al. proposed attribute reliability to reflect the objective reliability of data sources and did not optimize it together with other parameters^20^. In the online update model proposed by Zhou et al., attribute weights, rule weights, and belief degrees can be updated with newly generated data, but reference is not one of the online update options^21^. Therefore, the study of references should be separate from other parameters, in other words, the optimization for references and other parameters should be separated.

Precise values are usually used to express deterministic information, while expert knowledge contains uncertainty. Meanwhile, due to human preferences or conflicts of interest, this subjective approach always contains bias^22^. In addition, when the expert knowledge is insufficient, it will be difficult to determine the referential values accurately. Even if not bad modeling accuracy was achieved when random single-valued data were identified as references to antecedent attributes^23^, these referential values are still not interpretable, which is inconsistent with the idea of expert systems. Moreover, in group decision-making, experts may give various references. If these values cannot be represented completely, such as averaging them to obtain precise estimates, it will inevitably lead to the loss of some important information. In this context, if single-valued references are used as model parameters, their disadvantages will also be introduced into the model, resulting in the accuracy of the model being degraded. To preserve different references and facilitate further study and discussion, the problem of extending single-valued references to interval-valued references arises.

To integrate uncertain expert knowledge in the determination of the references, a new reference representation and optimization scheme is developed in the BRB-IR. In the BRB-IR, the alternatives of antecedents can be precise data or interval data. In this context, the uncertainty of expert knowledge in determining references can be fully expressed. In the processing of interval reference, an interval reference is transformed into multiple single-valued references, and then model parameters are transformed from interval form to single-valued form, which makes the inference procedures the same as the classical BRB. To better exploit the references provided by experts, a nonlinear optimization method is developed in which both qualitative information and quantitative data are employed. Therefore, the capability of the BRB expert system to express expert knowledge is enhanced, and the modeling accuracy is further improved.

This paper is organized as follows: In the "Brief presentation of BRB" section, the BRB expert system is briefly introduced. In the "Problem formulation and a new BRB-IR" section, the referential problems in the current BRB model are described, and then the BRB-IR is constructed. The implementation procedures of the BRB-IR are presented in the "Implementation of the BRB-IR" section. A case study of pipeline leak detection is conducted in the "Case study" section. This paper is concluded in the "Conclusion" section.

## Brief presentation of BRB

In this section, some basic definitions of BRB are presented, and the inference methodologies of the BRB are briefly introduced.

The classical BRB model consists of many belief rules to capture the nonlinear causal relationships between the antecedents and their associated consequents. Its $k_{th}$ rule is described as:

1 $$ \begin{gathered} R_{k} :{\text{ if }}x_{1} {\text{ is }}A_{1}^{k} \, \wedge \, x_{2} {\text{ is }}A_{2}^{k} \, \wedge \, \cdots \, \wedge \, x_{{M_{k} }} {\text{ is }}A_{{M_{k} }}^{k} , \hfill \\ {\text{then}} \{(D_{1} {, }\beta_{1,k} {),(}D_{2} {, }\beta_{2,k} {),} \cdots ,{(}D_{N} {, }\beta_{N,k} )\} (\sum\limits_{i = 1}^{N} {\beta_{i,k} \le 1} ), \hfill \\ {\text{ with a rule weight }}\theta_{k} , \hfill \\ {\text{ and attribute weights }}\delta_{k1} ,\delta_{k2} , \cdots ,\delta_{{kM_{k} }} , \hfill \\ \, k \in \{ 1, \cdots ,L\} \hfill \\ \end{gathered} $$

where $R_{k}$ denotes the $k_{th}$ rule in the BRB model, $x_{1} ,x_{2} , \cdots ,x_{{M_{k} }}$ represent the antecedents used in the $k_{th}$ rule, $A_{1}^{k} ,A_{2}^{k} , \cdots ,A_{{M_{k} }}^{k}$ and $\delta_{k1} ,\delta_{k2} , \cdots ,\delta_{{kM_{k} }}$ are the corresponding references and attribute weights of each antecedent in the $k_{th}$ rule, respectively. $D_{1} ,D_{2} , \cdots ,D_{n}$ and $\beta_{1,k} ,\beta_{2,k} , \cdots ,\beta_{n,k}$ are the consequents and their belief degrees in the $k_{th}$ rule, respectively. $M_{k}$ and $N$ represent the attribute number and consequent number in the $k_{th}$ rule, respectively. $\theta_{k}$ is the rule weight of the $k_{th}$ rule, $L$ is the number of rules in the BRB.

The rule base is first established, and then the evidential reasoning (ER) approach is utilized to aggregate the activated rules^24^. As two widely used inference engines, the recursive ER and analytical ER were proposed by Yang et al. in 2006 and 2007, respectively^2,25^. The former can describe the aggregation process clearly, and the latter can optimize the model parameters^26^. Therefore, these two methods have different suitable conditions, and they can be utilized in different procedures or different models^27,28^.

## Problem formulation and a new BRB-IR

In this section, the problem of single-valued referential values in practice is formulated in the "Problem formulation" section, and the BRB-IR model is developed in the "New BRB-IR" section.

### Problem formulation

The single-valued references may be affected by the bias of the expert individuals, group decision-making, and insufficient expert knowledge. The three disturbance factors are outlined as follows:

1. The expert's bias or preference: The BRB expert system is constructed based on the domain expertise and preferences of human experts. Due to the different preferences of experts and different analyses of the problem, the given model parameters are naturally different. In addition, with the increment or improvement of the knowledge, the single-valued references provided by the experts may fluctuate.
2. Group decision-making: Group decision-making means that there is more than one person to make decisions. In this case, people are influenced by others when making decisions^29^. Due to the different levels of domain knowledge, the decisions made by experts from the group have a certain difference. However, the single-valued references can only address precise information, thus they are not applicable in this situation.
3. Insufficient expert knowledge: Sometimes, expert knowledge is inadequate in some fields. For example, in cutting-edge medical research, it is difficult to describe indicators accurately. The uncertainty of qualitative information cannot be developed in the model since the single-valued references only address deterministic information, which will degrade the accuracy of the model.

It can be seen from the above analysis that the current reference expression method cannot fully represent expert knowledge and has a certain extent of loss of information. In this case, to improve the BRB's information representation ability and its modeling accuracy, it is crucial to express referential values given by experts more rationally and process them in the model effectively. Therefore, a BRB model with the capability to deal with imprecise referential information needs to be proposed.

### New BRB-IR

To express uncertain expert knowledge in references, a BRB model with interval-valued references (BRB-IR) is proposed, and its $k{\text{th}}$ rule is described as:

2 $$ \begin{gathered} R_{k} :{\text{ if }}x_{1} {\text{ in [}}A_{1}^{k - } {, }A_{1}^{k + } {] } \wedge \, \cdots \, \wedge \, x_{{M_{k} }} {\text{ in [}}A_{{M_{k} }}^{k - } {, }A_{{M_{k} }}^{k + } {]}, \hfill \\ {\text{then}} \{ (D_{1} {, }\beta_{1,k} {),} \cdots ,{(}D_{N} {, }\beta_{N,k} )\} (\sum\limits_{i = 1}^{N} {\beta_{i,k} \le 1} {),} \hfill \\ {\text{ with a rule weight }}\theta_{k} , \hfill \\ {\text{ and attribute weights }}\delta_{k1} , \cdots ,\delta_{{kM_{k} }} , \hfill \\ \, k \in \{ 1, \cdots ,L\} \hfill \\ \end{gathered} $$

where $[A_{i}^{k - } , \, A_{i}^{k + } ], \, (i = 1, \cdots ,M_{k} )$ denote the interval-valued references of the $i{\text{th}}$ attribute in the $k{\text{th}}$ rule, $A_{i}^{k - }$ and $A_{i}^{k + }$ are the lower bound and upper bound of the interval, respectively. It can be drawn that when all references are changed to a single-valued form, the BRB-IR will transform to be the classical BRB in which the references are confirmed by experts without any ignorance.

As an extension of single-valued references, interval-valued references are developed for better exploitation of uncertain information. They can better represent the situations in which expert knowledge is insufficient and better reflect the expert knowledge's vagueness and roughness in group decision-making. Moreover, the interval form is a better representation method of expertise than the precise form since the interval value has better tolerance of faults than the precise value.

In BRB-IR, the initial referential values are interval-valued data given by experts, and then an optimization algorithm is applied to optimize them by integrating interval references and data samples. Therefore, the optimized references are determined by considering qualitative knowledge and quantitative data. As a result, the obtained referential values are the optimal references of the antecedent attributes in BRB-IR.

Although the interval references are given by experts, which means they also have bias and uncertainty, the interval-valued data can better represent expert knowledge than single-valued data. Moreover, as long as the interval value covers the optimal reference, it can be obtained in the optimization stage. In other words, compared to the single-valued reference, the interval reference can reduce the bias of the experts but cannot eliminate the bias and uncertainty.

Theoretically, the referential value of the consequence can also be extended to an interval form since it is also determined by experts. However, its processing method is the same as that of the antecedent attribute. Therefore, in this paper, to simplify the problem while still presenting the interval reference processing method completely, only the reference of the antecedent attribute is extended to the interval form.

## Implementation of the BRB-IR

In this section, the modeling procedure of the BRB-IR is presented.

### Process of the interval-valued references

**Figure 1:**
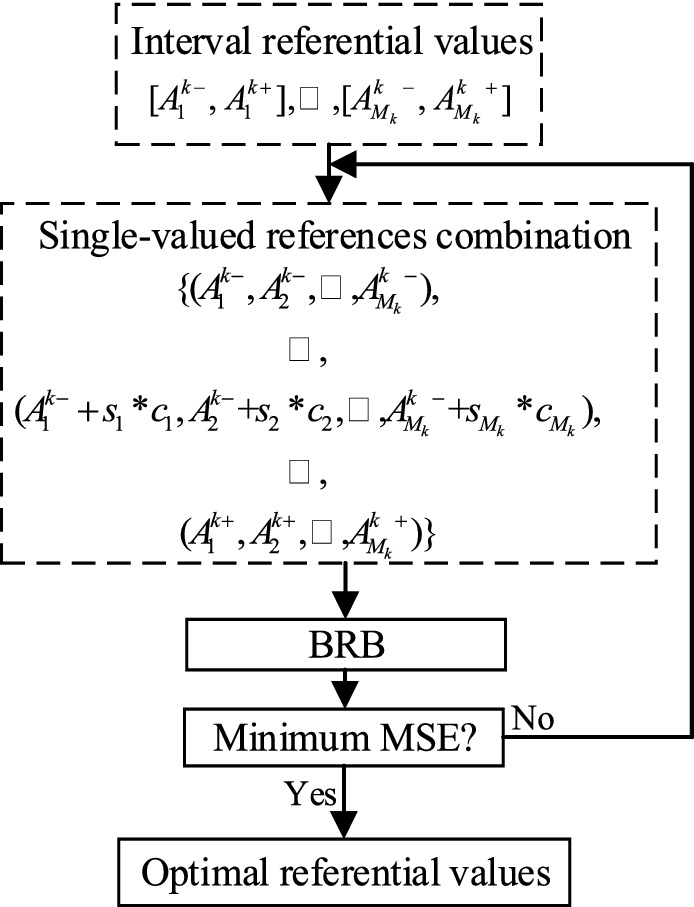
Calculation process of the nonlinear optimization algorithm.

In the BRB-IR, the initial referential values are provided by experts. Then optimized by a nonlinear optimization algorithm (NOA) defined in Eq. (3), the optimization objective is to obtain the minimum mean square error (MSE) of the BRB. The optimization process is illustrated in Fig. 1.

3 $$ \begin{gathered} {\text{min}}\,\,\,MSE(A_{i}^{k} ) \hfill \\ st. \hfill \\ A_{i}^{k - } \le A_{i}^{k} \le A_{i}^{k + } , \, i = 1, \ldots ,M_{k} \hfill \\ \end{gathered} $$

where $A_{i}^{k-}$ and $A_{i}^{k+}$ denote the lower bound and upper bound of the references of the $i{\text{th}}$ attribute, respectively, and they are provided by experts. $MSE(A_{i}^{k} )$ is calculated as:

4 $$ MSE(A_{i}^{k} ) = \frac{1}{T}\sum\limits_{t = 1}^{T} {(BRB_{estimated} - BRB_{actual} )^{2} } $$

where $T$ is the number of input data, $output_{estimated}$ and $output_{actual}$ are the estimated and actual output of the BRB, respectively.

As shown in Fig. 1, $s_{i} (i = 1, \cdots ,M_{k} )$ represent the step size of the $i_{th}$ attribute's reference, and $c_{i} (i = 1, \cdots ,M_{k} )$ correspond to the number of steps. To obtain all the single-valued combinations, the single value of each attribute is needed to be selected first. The lower bound and upper bound of each interval have been given, then set a step size, and all the single values in the interval can be obtained. For example, if the initial interval references of the two attributes $\{ x_{1} ,x_{2} \}$ are [1.6, 1.8] and [0.5, 0.7] respectively, and the step sizes are 0.0001 and 0.0002 respectively. Then the single-valued combinations are {(1.6, 0.5),(1.6, 0.5 + 0.0002*1),…, (1.6, 0.7), (1.6 + 0.0001*1, 0.5), (1.6 + 0.0001*1, 0.5 + 0.0002*1),…, (1.8, 0.5), (1.8, 0.5 + 0.0002*1),…, (1.8, 0.7)}.

After all the single-valued combinations are obtained, the value in each combination corresponds to the combination of attribute referential values, which can be shown in Table 1.

**Table 1:** Single-valued combinations for each attribute.

${{x}}_{{1}}$	1.6	1.6	…	1.6	1.6 + 0.0001*1	1.6 + 0.0001*1	…	1.8	1.8	…	1.8
${\text{x}}_{{2}}$	0.5	0.5 + 0.0002*1	…	0.7	0.5	0.5 + 0.0002*1	…	0.5	0.5 + 0.0002*1	…	0.7

In the nonlinear optimization process, the interval-valued references are first combined into a series of single-valued reference combinations. Then, each of the combinations is selected as the reference of the antecedent attributes in the BRB model. Then, every combination has a corresponding output of the model, and their MSEs can be calculated subsequently. Finally, the combination with the minimum MSE is picked, and those values are the optimal references for the BRB-IR. In other words, even if there is a smaller MSE when only quantitative data is considered in the model, it is not a reasonable result since it cannot meet the requirements of experts.

In general, the interval values given by experts are not modified in modeling. Therefore, as the step size decreases, the number of combinations will increase accordingly, and the interval referential value will be more fully introduced to the model, thus the obtained reference will be increasingly closer to the ideal reference. The step size is a trade-off between optimization accuracy and computational complexity, and it can be determined in two ways: (1) Based on the number of points needed in the interval. For example, if $n$ points need to be generated from $[lb,ub]$, then the step size is $(ub - lb)/n$. The number $n$ is provided by experts, and further adjustments can be made when the modeling accuracy requirements are not met after one round of calculations. (2) Based on the accuracy requirement of the decimal. For example, if the accuracy requirement is 3 digits after the decimal point, then the step size can be set to 0.001.

The final referential values obtained have taken expert knowledge and quantitative data into consideration, in which expert knowledge refers to the interval-valued references given by the expert, it defines the boundary of each reference, and the quantitative data refers to the sample data in the optimization process.

### Reasoning of the BRB-IR

Once the optimal referential values are obtained, the model can be reasoned by the following processes:

#### Input transformation

Based on the different natures of the attributes, the transformation includes the transformation of qualitative attributes, quantitative attributes, and symbolic attributes^2^. Among the transformation methods of quantitative transformation, the utility-based equivalence transformation method can preserve the features of original assessments and is suitable for decision analysis under uncertainties^30^. It can be described by:

5 $$ a_{i}^{k} = \left\{ \begin{gathered} \frac{{A_{i}^{l + 1} - x_{i} }}{{A_{i}^{l + 1} - A_{i}^{l} }}, \, k = l,A_{i}^{l} \le x_{i} \le A_{i}^{l + 1} \hfill \\ \hfill \\ 1 - a_{i}^{k} , \quad k = l + 1 \hfill \\ 0, \quad k = 1, \ldots ,L, \, k \ne l,l + 1 \hfill \\ \end{gathered} \right. $$

where $a_{i}^{k}$ is the matching degree to the $i_{th}$ attribute, $x_{i}$ denotes the sample data, and $A_{i}^{l}$ is the reference of the $i_{th}$ attribute in the $l_{th}$ rule, which is calculated by the NOA.

#### Calculation of the activation weight

6 $$ \omega_{k} = \frac{{\theta_{k} \prod\limits_{i = 1}^{{M_{k} }} {(a_{i}^{k} )^{{\overline{{\delta_{i} }} }} } }}{{\sum\limits_{l = 1}^{L} {\theta_{l} } \prod\limits_{i = 1}^{{M_{k} }} {(a_{i}^{l} )^{{\overline{{\delta_{i} }} }} } }} $$

7 $$ \overline{{\delta_{i} }} = \frac{{\delta_{i} }}{{\mathop {\max }\limits_{{i = 1, \cdots ,M_{k} }} \{ \delta_{i} \} }} $$

where $\omega_{k}$ denotes the activation weight of the $k_{th}$ rule, $\overline{{\delta_{i} }}$ is the normalized attribute weight of the $i_{th}$ attribute.

#### Calculation of the final belief degree by the analytical ER algorithm

8 $$ \mu = [\sum\limits_{n = 1}^{N} {\prod\limits_{k = 1}^{L} {(\omega_{k} \beta_{n,k} + 1 - \omega_{k} \sum\limits_{j = 1}^{N} {\beta_{j,k} } ) - (N - 1)\prod\limits_{k = 1}^{L} {(1 - \omega_{k} \sum\limits_{j = 1}^{N} {\beta_{j,k} } )} } } ]^{ - 1} $$

9 $$ \beta_{n} = \frac{{\mu [\prod\limits_{k = 1}^{L} {(\omega_{k} \beta_{n,k} + 1 - \omega_{k} \sum\limits_{j = 1}^{N} {\beta_{j,k} } ) - \prod\limits_{k = 1}^{L} {(1 - \omega_{k} \sum\limits_{j = 1}^{N} {\beta_{j,k} } )} } ]}}{{1 - \mu [\prod\limits_{k = 1}^{L} {(1 - \omega_{k} )} ]}} $$

where $\beta_{n}$ denotes the belief degree in the final belief distribution.

#### Utility calculation

After aggregating all the rules, the output of the BRB-IR is expressed as:

10 $$ S(x) = \{ (D_{n} , \, \beta_{n} );n = 1, \cdots ,N\} $$

Let $\mu (D_{n} )$ represents the utility of $D_{n}$, then the expected utility of $S(x)$ is:

11 $$ u(S(x)) = \sum\limits_{n = 1}^{N} {u(D_{n} )\beta_{n} } $$

According to the above analysis, the basic modeling procedures of the BRB-IR are described as:

Step 1: Obtain the interval-valued references given by experts.
Step 2: The optimal referential values are calculated by the NOA proposed in the "Process of the interval-valued references" section.
Step 3: Calculate the matching degrees using Eq. (5).
Step 4: Calculate the activation weights using Eqs. (6) and (7).
Step 5: The activated rules are aggregated by the ER approach using Eqs. (8) and (9).
Step 6: Calculate the final output of the BRB-IR according to utility theory.

##### Remark 1

According to different scenarios, the size of the BRB may increase exponentially. To better apply the interval reference to conjunctive BRB, if a rule explosion occurs, then it is necessary to perform the rule reduction first and then apply it to the reduced BRB. The application of interval reference in the disjunctive BRB will be further studied in future work.

### Optimization of the BRB-IR

To optimize the remaining parameters of the model, including rule weights, attribute weights, and belief degrees, the objective function is constructed as:

12 $$ {\text{min}}\;\;\;MSE(\theta_{k} , \, \beta_{n,k} , \, \delta_{i} ) $$

where $MSE(\theta_{k} , \, \beta_{n,k} , \, \delta_{i} )$ can measure the accuracy of the model, which is calculated as:

13 $$ MSE(\theta_{k} , \, \beta_{n,k} , \, \delta_{i} ) = \frac{1}{T}\sum\limits_{t = 1}^{T} {(output_{estimated} - output_{actual} )^{2} } $$

where $T$ is the number of samples, $output_{actual}$ and $output_{estimated}$ are the actual and estimated output of the system, respectively, and the latter is calculated as:

14 $$ output_{estimated} = u(S(x)) = \sum\limits_{n = 1}^{N} {\mu (D_{n} )\beta_{n} } $$

To further present the optimization method of the BRB-IR, the model can be described as:

15 $$ \begin{gathered} {\text{min}}\,\,\,\,MSE(\theta_{k} , \, \beta_{n,k} , \, \delta_{i} ) \hfill \\ st. \hfill \\ 0 \le \theta_{k} \le 1, \, k{ = 1,} \cdots {,}L{,} \hfill \\ 0 \le \delta_{i} \le 1, \, i{ = 1,} \cdots {,}M_{k} {,} \hfill \\ 0 \le \beta_{n,k} \le 1, \, n = 1,...,N, \, k{ = 1,} \cdots {,}L, \hfill \\ \sum\limits_{n = 1}^{N} {\beta_{n,k} } = 1, \, k{ = 1,} \cdots {,}L \hfill \\ \end{gathered} $$

At present, many optimization algorithms are used to optimize the parameters of the original or variant BRB. Zhou et al. used the projection covariance matrix adaptation evolutionary strategy (P-CMA-ES), constrained particle swarm algorithm (PSO), and sequential quadratic programming (SQP) to optimize the hidden belief rule base with power set (PHBRB) respectively, and the results showed that the trained PHBRB has better modeling accuracy than the other two optimization methods^31^. Cao et al. compared the optimization of BRB by differential evolution algorithm (DE), P-CMA-ES, and PSO, and explained that P-CMA-ES guarantees the interpretability of the model while ensuring the optimization effect^3^. R. U. Islam studied the deterministic and non-deterministic methods of BRB optimization and enhanced the modeling capabilities of the model^32,33^.

As shown in Eq. (15), the optimization of the BRB belongs to single-objective multi-constraint optimization. In view of the superiority of P-CMA-ES on BRB optimization, this paper adopts it as the optimization algorithm, which includes the following steps:

Step 1: Parameter initialization.
  The initial parameter $w^{0} = \Omega^{0}$ denotes the parameters that need to be optimized, and $\Omega^{0}  = \{ \theta_{1} , \cdots ,\theta_{L} {,}\delta_{1} {,} \cdots {,}\delta_{{M_{k} }} {,}\beta_{1,1} {,} \cdots {,}\beta_{N,L} \}$.
Step 2: Obtain each generation by the sampling operation, which can be described in Eq. (16).
  16 $$ \Omega_{i}^{g + 1} \sim w^{g} + \varepsilon^{g} N(0,C^{g} ),i = 1, \cdots ,\lambda $$
  where $\Omega_{i}^{g + 1}$ represents the $i_{th}$ solution when it evolves to the $(g + 1)_{th}$ generation, $w^{g}$ and $\varepsilon^{g}$ denote the mean and step size of the $g_{th}$ generation respectively, $C^{g}$ is the covariance matrix of the $g_{th}$ generation, $N( * )$ represents the normal distribution, $\lambda$ denotes the number of offspring.
Step 3: Project the solution to the feasible hyperplane to satisfy the constraints of Eq. (17). The hyperplane can be represented as Eq. (18).
  17 $$ \begin{gathered} \Omega_{i}^{g + 1} (1 + n_{e} \times (j - 1):n_{e} \times j) = \Omega_{i}^{g + 1} (1 + n_{e} \times (j - 1):n_{e} \times j) - A_{e}^{T} \times (A_{e} \times A_{e}^{T} )^{ - 1} \hfill \\ \, \times \Omega_{i}^{g + 1} (1 + n_{e} \times (j - 1):n_{e} \times j) \times A_{e} \hfill \\ \end{gathered} $$
  18 $$ A_{e} \Omega_{i}^{g} (1 + n_{e} \times (j - 1):n_{e} \times j) = 1 $$
  where $A_{e} = [1, \cdots ,1]_{1 \times N}$ denotes the parameter vector, $n_{e} = (1, \cdots ,N)$ and $j = (1, \cdots ,N + 1)$ represent the number of constrained variables and equality constraints in solution $\Omega_{i}^{g}$, respectively.
Step 4: Update the mean of the next generation by Eq. (19).
  19 $$ w^{g + 1} = \sum\limits_{i = 1}^{\tau } {h_{i} \Omega_{i:\lambda }^{g + 1} } $$
  where $h_{i}$ represents the weight coefficient, $\Omega_{i:\lambda }^{g + 1}$ is the $i_{th}$ solution among the $\lambda$ solutions of the $(g + 1)_{th}$ generation, $\tau$ represents the offspring population size.
Step 5: Update the covariance matrix by Eq. (20).
  20 $$ C^{g + 1} = (1 - c_{1} - c_{2} )C^{g} + c_{1} P_{c}^{g + 1} \left( {P_{c}^{g + 1} } \right)^{T} + c_{2} \sum\limits_{i = 1}^{\nu } {h_{i} \left( {\frac{{K_{i:\lambda }^{g + 1} - \varphi^{g} }}{{\rho^{g} }}} \right)\left( {\frac{{K_{i:\lambda }^{g + 1} - \varphi^{g} }}{{\rho^{g} }}} \right)^{T} } $$
  where $\rho^{g}$ is the step size in the $g_{th}$ generation, $c_{1}$ and $c_{2}$ are learning rates, $P_{c}^{g + 1}$ denotes the evolution path of the $(g + 1)_{th}$ generation, $\varphi^{g}$ is the offspring population in the $g_{th}$ generation, $K_{i:\lambda }^{g + 1}$ represents the $i_{th}$ parameter vector from $\lambda$ vectors in the $(g + 1)_{th}$ generation.
Step 6: Execute *Step 1* to *Step 5* recursively until the optimal parameters are obtained.

### Modeling method of the complex system based on BRB-IR

In the implementation procedures of the BRB-IR for complex systems, there are three main steps: model construction, parameter training, and model testing^34^. These details are outlined as follows.

The first is the model construction. Based on the parameters given by experts and the observational data, the initial BRB-IR model is constructed in this part.

The initial parameters of the BRB-IR contain uncertainty since they are provided by experts. To reduce their influence on the modeling accuracy, they should be optimized first. In this part, they can be trained by the optimization process to deal with uncertainty. It should be noted that the references of all antecedents are calculated by the NOA, as presented in the "Process of the interval-valued references" section. The other parameters are optimized by the optimization process, as presented in the "Optimization of the BRB-IR" section.

The third is the testing part. In this part, the utility theory is utilized to calculate the final output of the BRB-IR, and the modeling accuracy of the model is tested.

**Figure 2:**
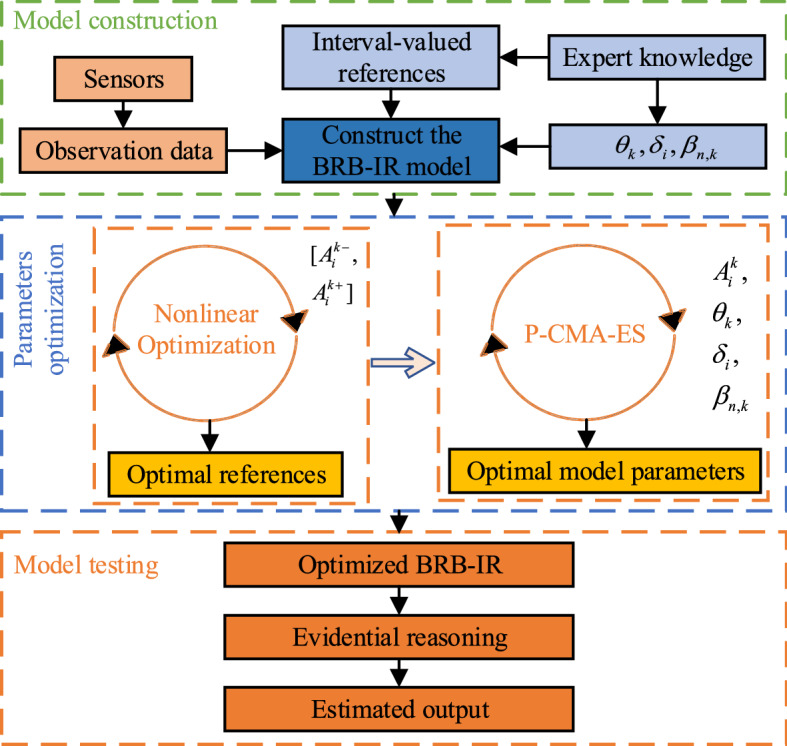
The implementation procedures of the BRB-IR.

The implementation of the BRB-IR is shown in Fig. 2 and outlined as follows:

Step 1 Obtain the dataset and divide it into training and testing samples.
Step 2 Construct the initial BRB-IR based on the data and parameters.
Step 3 Train the model parameters.
  Step 3.1 Obtain the optimal references by the method proposed in the "Process of the interval-valued references" section.
  Step 3.2 Train other parameters by the method presented in the "Optimization of the BRB-IR" section.
Step 4 Test the modeling accuracy.
  Step 4.1 Calculate the matching degrees and activation weights using Eqs. (5) and (7).
  Step 4.2 Aggregate the activated rules using the ER approach presented in Eqs. (8) and (9).
  Step 4.3 Calculate the output of the BRB-IR using Eq. (11).

## Case study

In this section, a case study for pipeline leak detection is presented to illustrate the effectiveness of the proposed BRB-IR model.

### Problem formulation of the pipeline leak detection

Pipeline leak detection is important and can not only prevent resource leaks but also prevent a series of adverse consequences, such as environmental pollution. In this paper, the pipeline leak detection introduced in^23^ is used in this experiment.

Under normal conditions of the pipeline, when inlet flow is larger (less) than outlet flow, the pressure in the pipeline will increase (decrease) since the total volume in the pipeline becomes larger (less). However, when this pattern is broken, such as when the inlet flow is increased and the pressure is decreased, then it is highly likely that the pipeline is leaking. Therefore, the *FlowDiff* which represents the flow difference between inlet and outlet, and *PressureDiff* which represents the average pipeline pressure change over time, are selected as the key indicators of the model. The corresponding *LeakSize* is regarded as the consequent attribute.

According to different research scenarios and requirements, the key indicators can be determined through data analysis or expert knowledge. After identifying key indicators and references that need to be expressed in interval form, the BRB-IR can be used to solve such interval reference problems.

**Figure 3:**
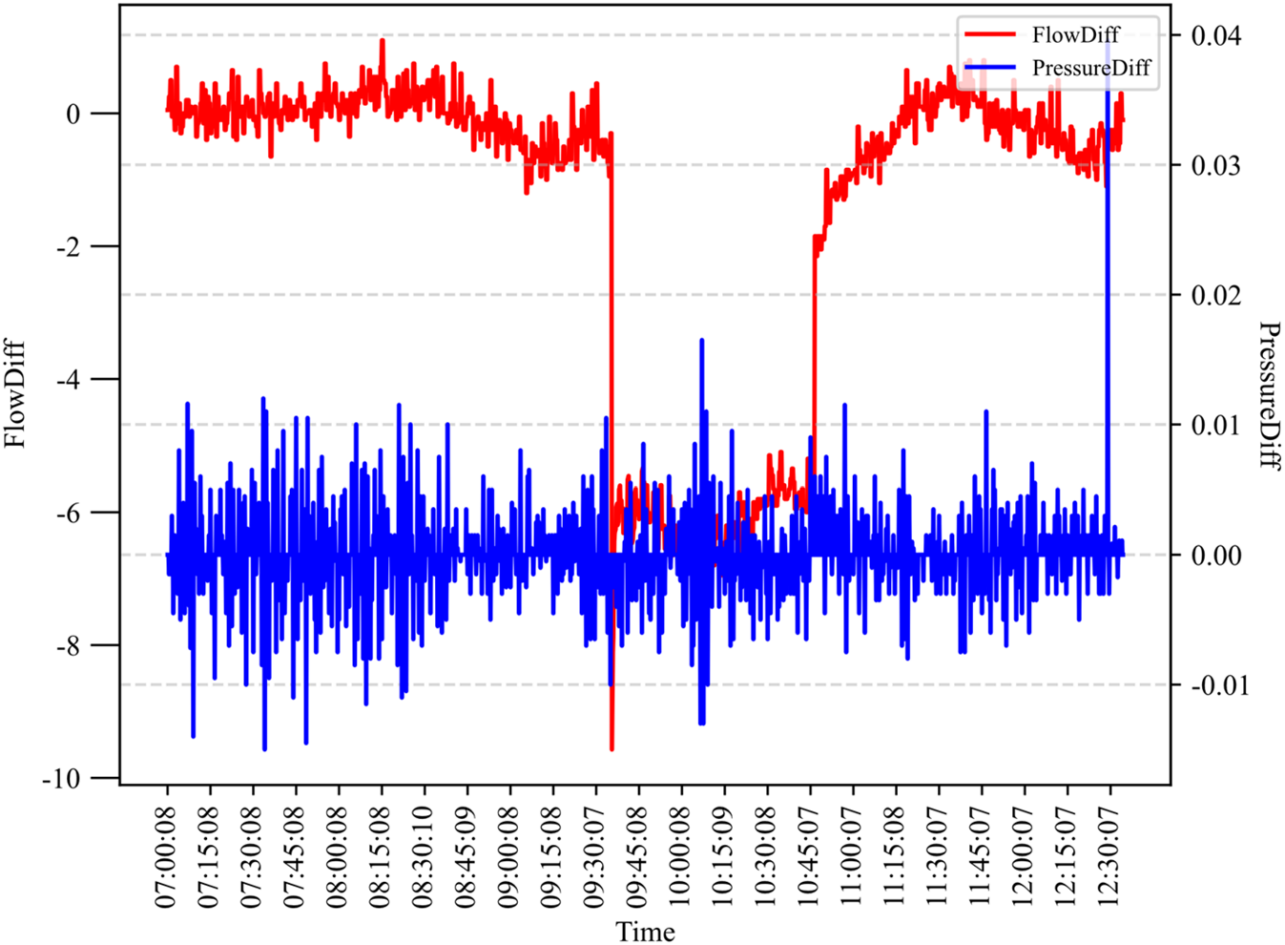
Calculated *FlowDiff* and *PressureDiff* of the samples.

In the pipeline leak detection introduced in^23^, there are 2008 samples in the dataset, as shown in Fig. 3, and 500 samples are used to train parameters, which were collected in the three periods: 7:00 to 7:33, 9:46 to 10:20, and 10:50 to 11:08. The initial parameters are provided by experts, and only a few samples are needed to optimize the model parameters, that is, these parameters have been optimized by expert knowledge during initialization.

### Construction of the BRB-IR

Although the initial referential values in the BRB-IR are in interval form, the size of the rule base is still decided by the number of interval references of all antecedents. The initial interval referential values of *FlowDiff* and *PressureDiff* are shown in Tables 2 and 3. The referential points of *LeakSize* are shown in Table 4. It is worth noting that this experiment assumes that some referential values are still in single-valued form. On the one hand, when more referential values are in interval form, the computational complexity will exponentially increase. On the other hand, when part of the attributes is in intervals, the effectiveness of this method can also be proved.

**Table 2:** The initial references of the *FlowDiff.*

Antecedent attribute	negative large (NL)	negative medium (NM)	negative small (NS)	negative very small (NVS)	Zero (Z)	positive small (PS)	positive medium (PM)	positive large (PL)
*FlowDiff*	− 10	[ − 6, − 4.5]	[ − 3.5, − 2.5]	− 1	0	1	2	3

**Table 3:** The initial references of the *PressureDiff.*

Antecedent attribute	NL	NM	NS	Z	PS	PM	PL
*PressureDiff*	− 0.01	− 0.005	− 0.002	0	0.002	0.005	0.01

**Table 4:** The references of the *LeakSize.*

Consequent attribute	Zero (Z)	very small (VS)	Medium (M)	High (H)	Very high (VH)
*LeakSize*	0	2	4	6	8

In the pipeline leak detection based on BRB-IR, the $k_{th}$ rule is described as:

21 $$ \begin{gathered} R_{k} :{\text{ if }}FlowDiff{\text{ in [}}A_{1}^{k - } {, }A_{1}^{k + } {] } \wedge \, PressureDiff{\text{ in [}}A_{2}^{k - } {, }A_{2}^{k + } {]}, \hfill \\ {\text{then}} \{ (Z, \beta_{1,k} {\text{),(VS, }}\beta_{2,k} {\text{),(M, }}\beta_{3,k} {)},({\text{H}}, \, \beta_{4,k} ),{\text{(VH, }}\beta_{5,k} )\} (\sum\limits_{i = 1}^{5} {\beta_{i,k} \le 1} {),} \hfill \\ {\text{ with a rule weight }}\theta_{k} , \hfill \\ {\text{ and attribute weights }}\delta_{k1} ,\delta_{k2} , \hfill \\ \, k \in \{ 1, \cdots ,56\} \, \hfill \\ \end{gathered} $$

where ${[}A_{1}^{k - } {, }A_{1}^{k + } {]}$ and ${[}A_{2}^{k - } {, }A_{2}^{k + } {]}$ are the interval references of *FlowDiff* and *PressureDiff*, respectively. According to Tables 1 and 2, the belief rules are generated, and their initial values given by expert^23^ are shown in Table S1 of the “Appendix”.

**Figure 4:**
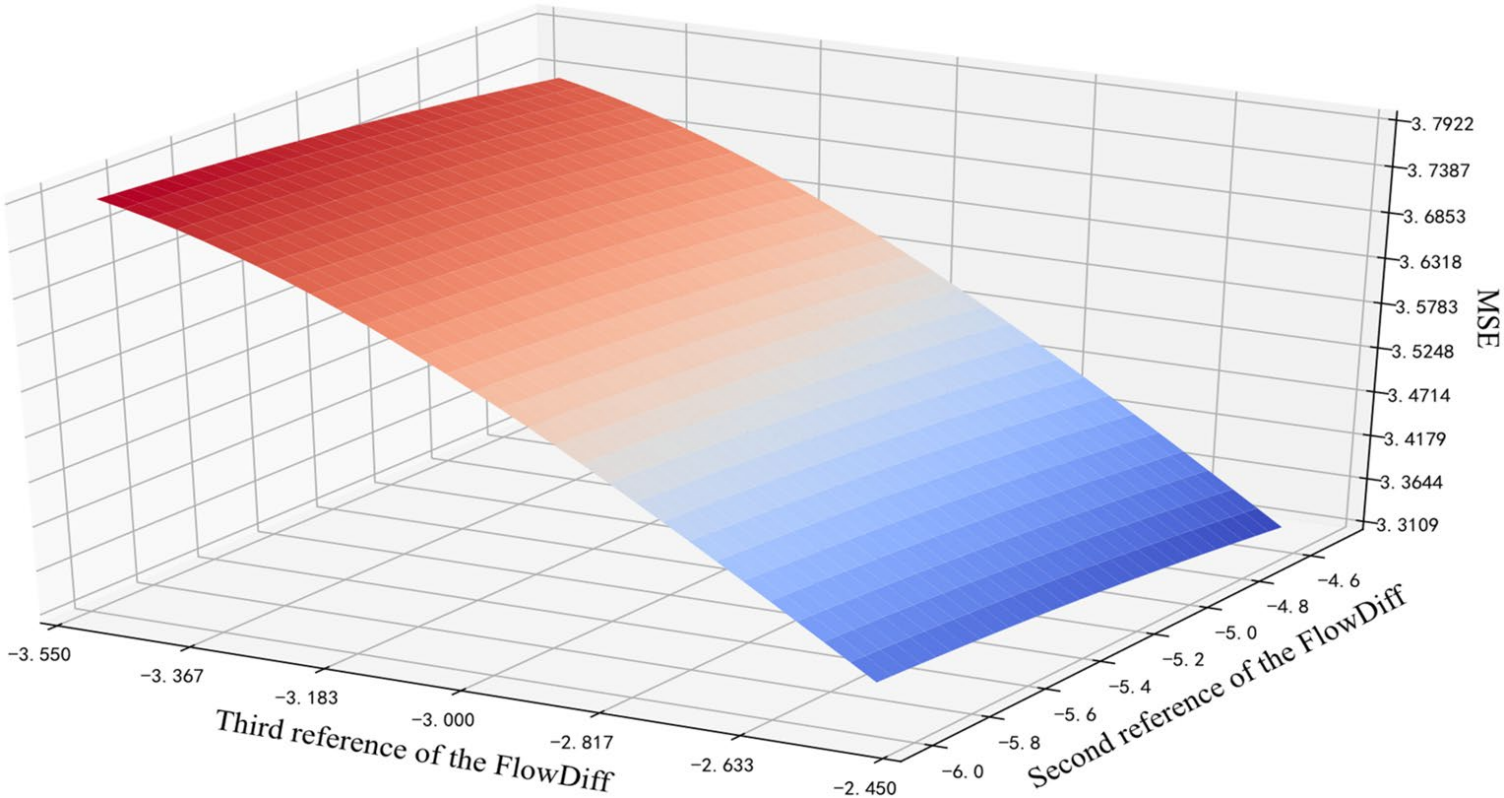
Calculated MSEs of the interval referential values in BRB.

After the initial references are provided, the MSEs of the BRB can be calculated by the NOA, and the results are shown in Fig. 4. The single-valued combinations have the corresponding MSEs, then the minimum MSE can be obtained, and its corresponding single-valued combination can be obtained simultaneously.

The optimal references of *FlowDiff* are shown in Table 5, and when combined with the singled-valued references in Table 3, the optimal references of the attributes are obtained. It is worth noting that the optimal referential values of *FlowDiff* in Table 5 are the optimal references only in the current situations. In other words, if more attributes are in interval form, these values may be changed.

**Table 5:** The optimal references of the *FlowDiff.*

Antecedent attribute	NL	NM	NS	NVS	Z	PS	PM	PL
*FlowDiff*	− 10	− 4.5	− 2.5	− 1	0	1	2	3

In the experiment, the remaining parameters except the belief degrees are set to 1. Then, the P-CMA-ES is utilized to optimize all the remaining parameters in which the population size and the generation number are 27 and 500, respectively.

The belief rules after optimization are shown in Table S2 of the “Appendix”, and the attribute weights of *FlowDiff* and *PressureDiff* are 0.9782 and 0.3763, respectively.

**Figure 5:**
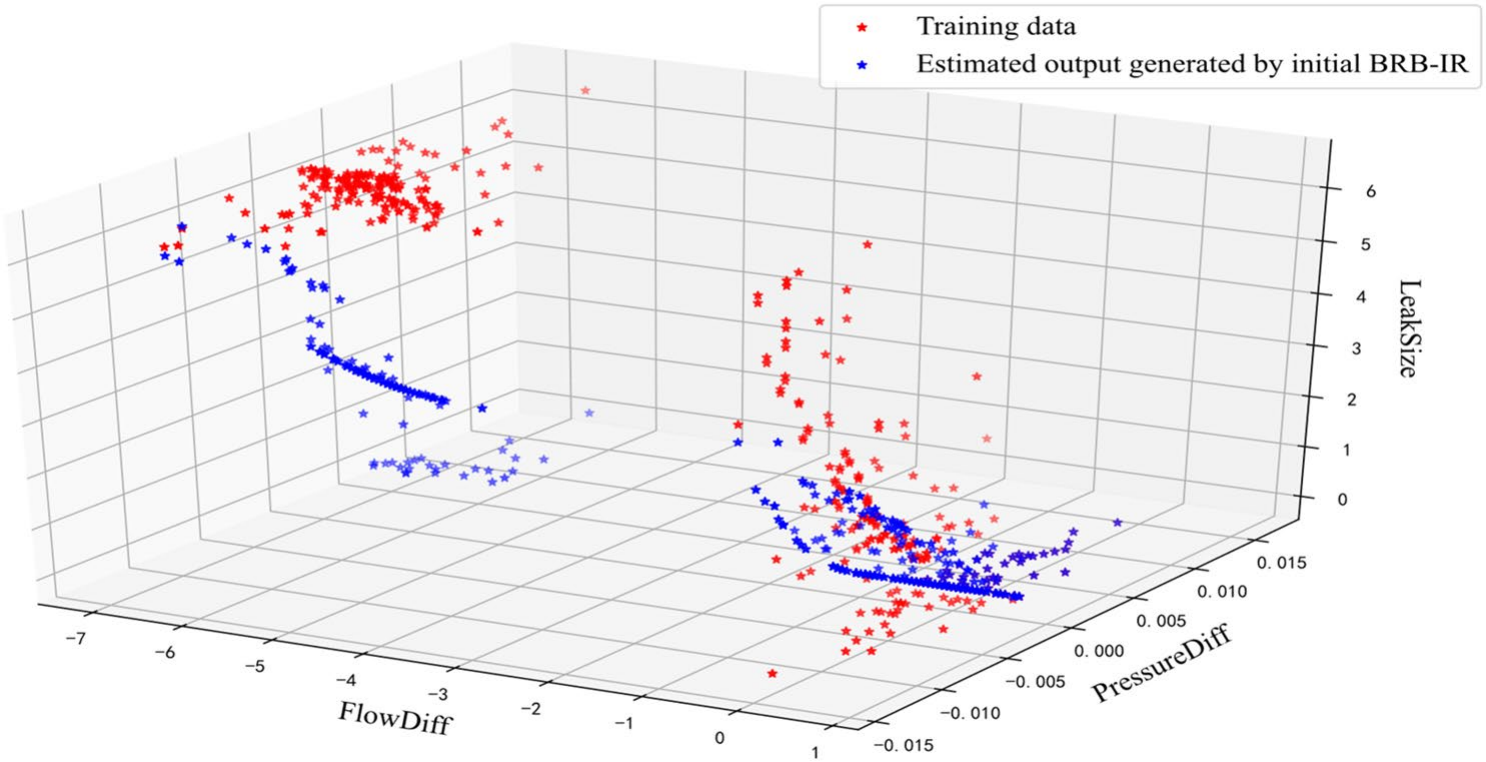
Estimated output of the initial BRB-IR.

**Figure 6:**
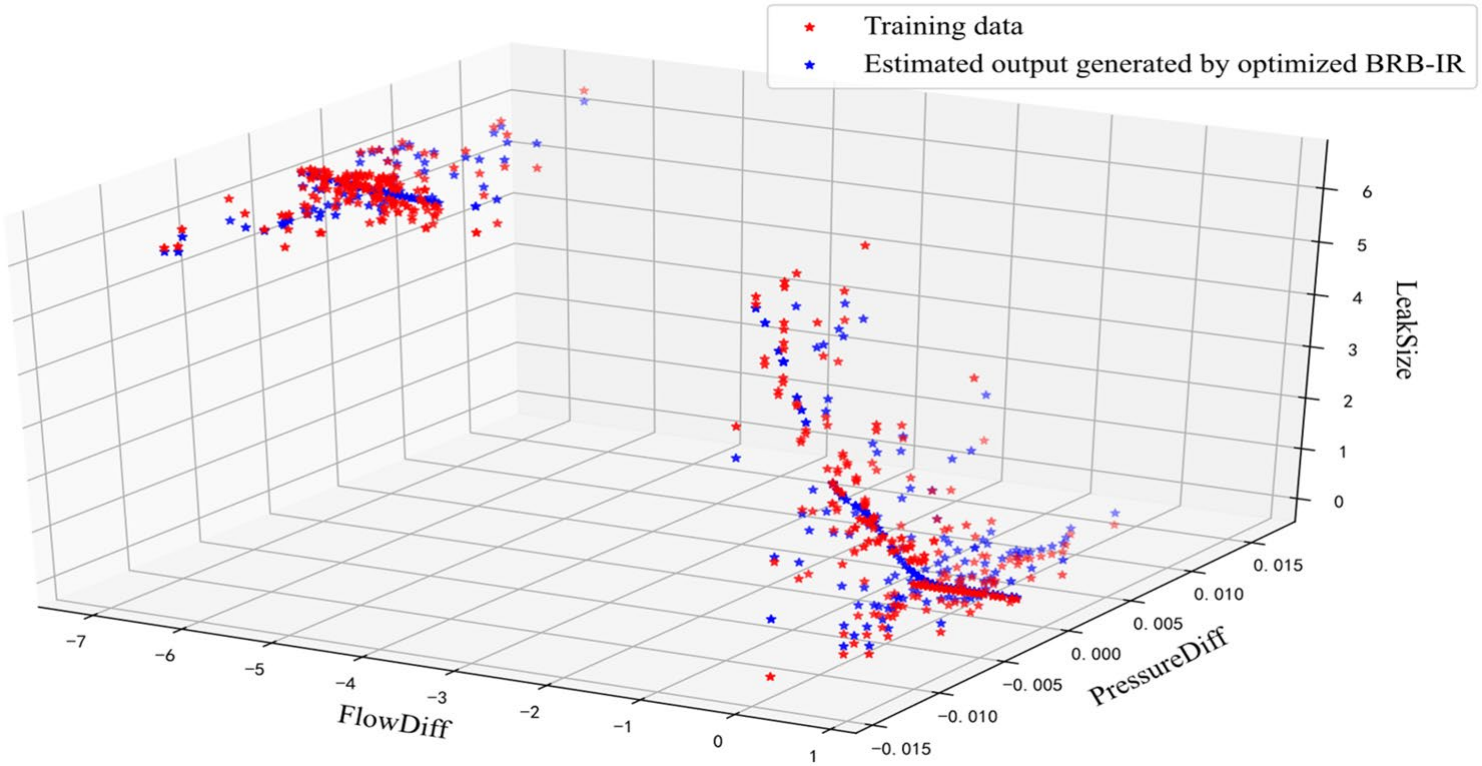
Estimated output of the optimized BRB-IR.

To illustrate the effectiveness of the optimization, the estimated output of the initial BRB-IR and optimized BRB-IR are shown in Figs. 5 and 6, respectively. It can be seen that the optimized BRB-IR can better detect the leakage of the pipeline than the initial BRB-IR.

### Comparative studies

The proposed BRB-IR is valid in leakage detection. To further verify the model's superiority, comparative studies between the BRB in^23^, fuzzy expert system, back propagation (BP), and the proposed BRB-IR are presented in this subsection.

By removing the optimization part of the BRB in^23^, the fuzzy expert system is obtained. Its belief rules are shown in Table S1 of the "Appendix".

**Figure 7:**
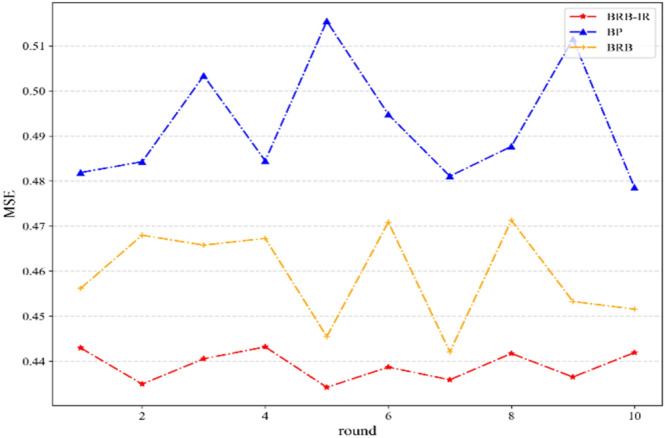
MSE comparison of the BRB-IR, BP, and BRB.

Ten rounds of tests are conducted, and the MSE means of all the methods are shown in Table 6. The results of each round of MSE for BP, BRB, and BRB-IR are shown in Fig. 7.

**Table 6:** The MSE means of the comparative methods.

Method	MSE
BRB-IR	0.4391
BRB	0.4592
Fuzzy Expert System	3.5244
BP	0.4923

As shown in Table 6, compared with the BRB, fuzzy expert system, and BP, the MSE of the BRB-IR improves by 4.4%, 87.54%, and 5.32% with only two referential values in interval form, respectively.

As shown in Fig. 7, it can be seen that the proposed BRB-IR can better detect leakage than BP and BRB, which illustrates the effectiveness of the BRB-IR.

When more single-valued references are replaced by interval-valued references, the qualitative information about the references will be more completely presented, and the modeling accuracy of the BRB-IR will certainly be further improved, but the computational complexity will also increase simultaneously. There are two ways to solve this problem: (1) Reduce the number of referential values, that is, perform parameter learning^18^. (2) Reduce the number of points in the interval that need to be acquired, which is also a trade-off between modeling accuracy and computational complexity.

**Figure 8:**
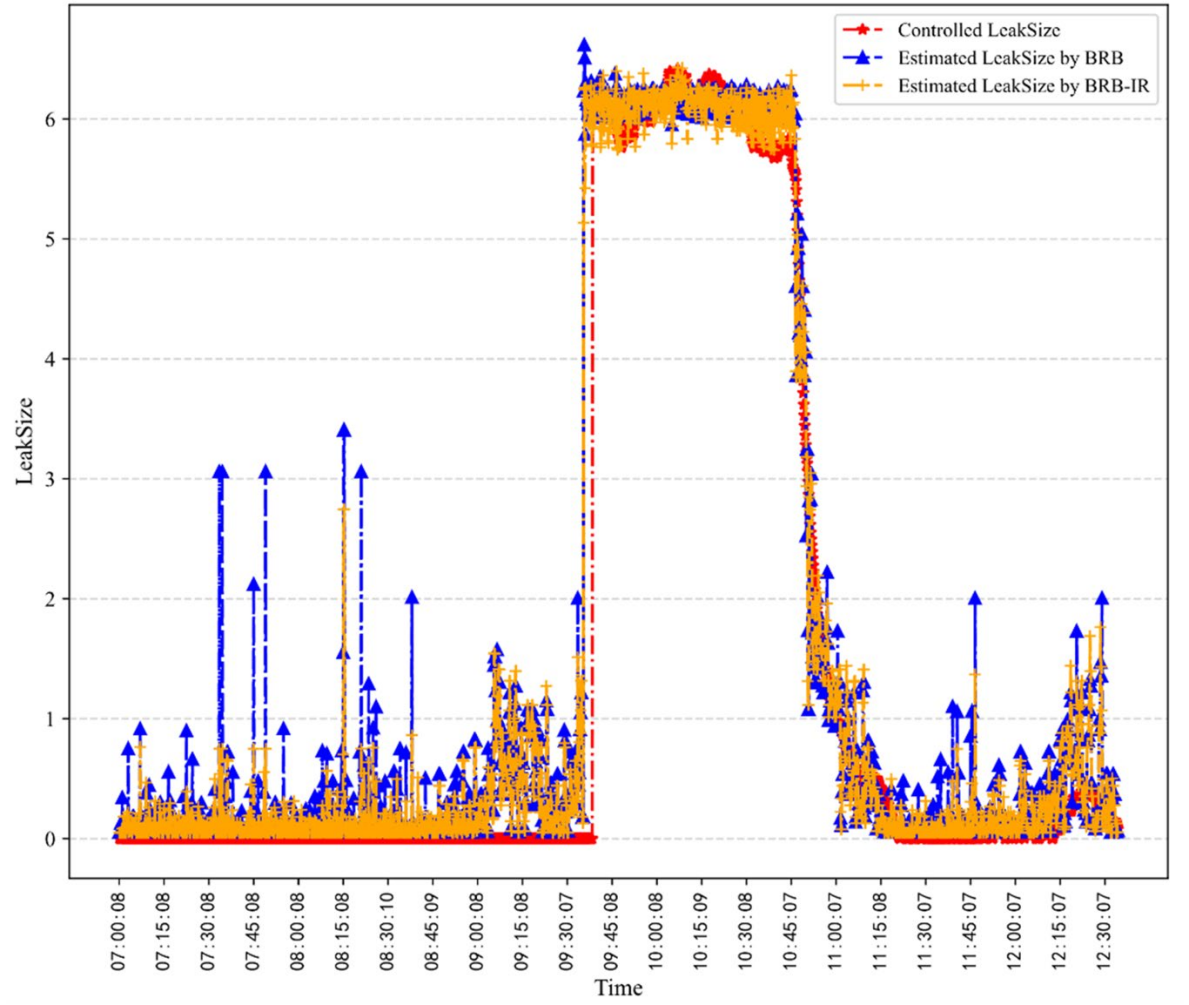
Estimated *LeakSize* by the classical BRB and BRB-IR.

To further show the superiority of the BRB-IR, the outputs of the classical BRB and the proposed BRB-IR are shown in Fig. 8. It can be seen that the BRB-IR has improved the modeling ability of the BRB and can detect leaks more accurately.

In the meanwhile, according to the comparative studies, the following conclusions can be drawn.

1. The MSEs of the BRB-IR were between 0.4343 and 0.4432 with a variance of 7.9901E-05, which verifies the robustness of the P-CMA-ES.
2. The BRB-based models have a clear causal relationship, thus the BRB-based leak detection method has better credibility than the data-driven method.
3. Data-driven methods, such as BP, need a large amount of data to train the parameters. It can be seen from the results that the BRB-based methods have superiority under small samples.
4. The parameters of the BRB-based method can be trained through optimization algorithms, thus the method that considers qualitative knowledge and quantitative data has better modeling accuracy than the method that only considers qualitative knowledge.

## Conclusion

In this paper, the current referential expression problems are formulated, and then a new BRB-IR is proposed. Due to the ignorance and vagueness of expert knowledge, the single-valued referential values in the classical BRB model cannot address these uncertainties rationally. In the BRB-IR, interval-valued references are used to address imprecise expert knowledge to further improve its knowledge representation ability and modeling accuracy, and the optimization of references is separate from the optimization of other parameters to provide an optional reference processing method. A case study of pipeline leak detection is presented to verify the effectiveness of the proposed BRB-IR.

The interval referential value is introduced into the BRB, and its modeling process has been fully demonstrated. Benefiting from the independent optimization of referential values, the selection of the optimization algorithm for reference will be more flexible. Moreover, the proposed reference processing method makes it convenient to be embedded into other existing BRBs and their variant models since it is decoupled from other parameter procedures. The interval-valued reference extends the representation ability of expert knowledge and improves the fault tolerance of the model. According to different modeling requirements, the original BRB and the new BRB-IR can be reasonably selected.

Based on the studies in this paper, future work on the BRB-IR can be carried out from the following aspects.

1. Introduce a better optimization algorithm to optimize the interval referential values and reduce the computational complexity.
2. Add IR to IRIMER to enhance IRIMIER's modeling capabilities.
3. Introduce the reliability of expert knowledge to further reduce the influence of fuzzy expert knowledge in the model.
4. Apply interval reference to disjunctive BRB.

## Supplementary Information

Supplementary Information.
