# Supplementary material for: A novel belief rule base expert system with interval-valued references
Source: Sci Rep. 2022 Apr 26;12:6786. doi: 10.1038/s41598-022-10636-8 (PMC9042851; doi:10.1038/s41598-022-10636-8)
Supplement: Supplementary file 1 — Supplementary Information. [file 41598_2022_10636_MOESM1_ESM.docx]

**A novel belief rule base expert system with interval-valued references**

Chao Sun^1^, Ruohan Yang^2^, Wei He^1,3,*^ & Hailong Zhu^1,*^

^1^ Harbin Normal University, Harbin 150025, China

^2^ Northwestern Polytechnical University, Xi'an 710072, China

^3^ Rocket Force University of Engineering, Xi'an 710025, China

^*^ Corresponding author: Hailong Zhu (zhuhailong2018@vip.163.com) & Wei He (he_w_1980@163.com).

**Appendix**

| **Rule number** | | ***FlowDiff* and *PressureDiff*** | | ***LeakSize* distribution $\{D_{1},D_{2},D_{3},D_{4},D_{5}\}=\{0,2,4,6,8\}$** | |
| --- | --- | --- | --- | --- | --- |
| 1 | | NL AND NL | | {(D_1_,0), (D_2_,0), (D_3_,0), (D_4_,0), (D_5_,1)} | |
| 2 | | NL AND NM | | {(D_1_,0), (D_2_,0), (D_3_,0), (D_4_,0.3), (D_5_,0.7)} | |
| 3 | | NL AND NS | | {(D_1_,0), (D_2_,0), (D_3_,0.2),(D_4_,0.8), (D_5_,0)} | |
| 4 | | NL AND Z | | {(D_1_,0), (D_2_,0), (D_3_,0.8), (D_4_,0.2), (D_5_,0)} | |
| 5 | | NL AND PS | | {(D_1_,0.65), (D_2_,0.35), (D_3_,0), (D_4_,0), (D_5_,0)} | |
| 6 | | NL AND PM | | {(D_1_,0.85), (D_2_,0.15), (D_3_,0), (D_4_,0), (D_5_,0)} | |
| 7 | | NL AND PL | | {(D_1_,0.95), (D_2_,0.05), (D_3_,0), (D_4_,0), (D_5_,0)} | |
| 8 | | NM AND NL | | {(D_1_,0), (D_2_,0), (D_3_,0.1), (D_4_,0.9), (D_5_,0)} | |
| 9 | | NM AND NM | | {(D_1_,0), (D_2_,0), (D_3_,0.7), (D_4_,0.3), (D_5_,0)} | |
| 10 | | NM AND NS | | {(D_1_,0), (D_2_,0.7), (D_3_,0.3), (D_4_,0), (D_5_,0)} | |
| 11 | | NM AND Z | | {(D_1_,0), (D_2_,0.9), (D_3_,0.1), (D_4_,0), (D_5_,0)} | |
| 12 | | NM AND PS | | {(D_1_,0.8), (D_2_,0.2), (D_3_,0), (D_4_,0), (D_5_,0)} | |
| 13 | | NM AND PM | | {(D_1_,0.9), (D_2_,0.1), (D_3_,0), (D_4_,0), (D_5_,0)} | |
| 14 | | NM AND PL | | {(D_1_,0.99), (D_2_,0.01), (D_3_,0), (D_4_,0), (D_5_,0)} | |
| 15 | | NS AND NL | | {(D_1_,0), (D_2_,0), (D_3_,0.4), (D_4_,0.6), (D_5_,0)} | |
| 16 | | NS AND NM | | {(D_1_,0), (D_2_,0), (D_3_,0.8), (D_4_,0.2), (D_5_,0)} | |
| 17 | | NS AND NS | | {(D_1_,0), (D_2_,0.3), (D_3_,0.6), (D_4_,0.1), (D_5_,0)} | |
| 18 | | NS AND Z | | {(D_1_,0.1), (D_2_,0.7), (D_3_,0.2), (D_4_,0), (D_5_,0)} | |
| 19 | | NS AND PS | | {(D_1_,0.7), (D_2_,0.3), (D_3_,0), (D_4_,0), (D_5_,0)} | |
| 20 | | NS AND PM | | {(D_1_,0.9), (D_2_,0.1), (D_3_,0), (D_4_,0), (D_5_,0)} | |
| 21 | | NS AND PL | | {(D_1_,1), (D_2_,0), (D_3_,0), (D_4_,0), (D_5_,0)} | |
| 22 | | NVS AND NL | | {(D_1_,0), (D_2_,0.1), (D_3_,0.4), (D_4_,0.5), (D_5_,0)} | |
| 23 | | NVS AND NM | | {(D_1_,0), (D_2_,0.8), (D_3_,0.2), (D_4_,0), (D_5_,0)} | |
| 24 | | NVS AND NS | | {(D_1_,0.2), (D_2_,0.7), (D_3_,0.1), (D_4_,0), (D_5_,0)} | |
| 25 | | NVS AND Z | | {(D_1_,1), (D_2_,0), (D_3_,0), (D_4_,0), (D_5_,0)} | |
| 26 | | NVS AND PS | | {(D_1_,1), (D_2_,0), (D_3_,0), (D_4_,0), (D_5_,0)} | |
| 27 | | NVS AND PM | | {(D_1_,1), (D_2_,0), (D_3_,0), (D_4_,0), (D_5_,0)} | |
| 28 | | NVS AND PL | | {(D_1_,1), (D_2_,0), (D_3_,0), (D_4_,0), (D_5_,0)} | |
| 29 | | Z AND NL | | {(D_1_,0), (D_2_,0.4), (D_3_,0.6), (D_4_,0), (D_5_,0)} | |
| 30 | | Z AND NM | | {(D_1_,0.2), (D_2_,0.7), (D_3_,0.1), (D_4_,0), (D_5_,0)} | |
| 31 | | Z AND NS | | {(D_1_,0.4), (D_2_,0.6), (D_3_,0), (D_4_,0), (D_5_,0)} | |
| 32 | | Z AND Z | | {(D_1_,1), (D_2_,0), (D_3_,0), (D_4_,0), (D_5_,0)} | |
| 33 | | Z AND PS | | {(D_1_,1), (D_2_,0), (D_3_,0), (D_4_,0), (D_5_,0)} | |
| 34 | | Z AND PM | | {(D_1_,1), (D_2_,0), (D_3_,0), (D_4_,0), (D_5_,0)} | |
| 35 | | Z AND PL | | {(D_1_,1), (D_2_,0), (D_3_,0), (D_4_,0), (D_5_,0)} | |
| 36 | | PS AND NL | | {(D_1_,0), (D_2_,0.8), (D_3_,0.2), (D_4_,0), (D_5_,0)} | |
| 37 | | PS AND NM | | {(D_1_,0.8), (D_2_,0.2), (D_3_,0), (D_4_,0), (D_5_,0)} | |
| 38 | | PS AND NS | | {(D_1_,0.95), (D_2_,0.05), (D_3_,0), (D_4_,0), (D_5_,0)} | |
| 39 | | PS AND Z | | {(D_1_,1), (D_2_,0), (D_3_,0), (D_4_,0), (D_5_,0)} | |
| 40 | | PS AND PS | | {(D_1_,1), (D_2_,0), (D_3_,0), (D_4_,0), (D_5_,0)} | |
| 41 | | PS AND PM | | {(D_1_,1), (D_2_,0), (D_3_,0), (D_4_,0), (D_5_,0)} | |
| 42 | | PS AND PL | | {(D_1_,1), (D_2_,0), (D_3_,0), (D_4_,0), (D_5_,0)} | |
| 43 | | PM AND NL | | {(D_1_,0.1), (D_2_,0.9), (D_3_,0), (D_4_,0), (D_5_,0)} | |
| 44 | | PM AND NM | | {(D_1_,0.3), (D_2_,0.7), (D_3_,0), (D_4_,0), (D_5_,0)} | |
| 45 | | PM AND NS | | {(D_1_,0.85), (D_2_,0.15), (D_3_,0), (D_4_,0), (D_5_,0)} | |
| 46 | | PM AND Z | | {(D_1_,0.98), (D_2_,0.02), (D_3_,0), (D_4_,0), (D_5_,0)} | |
| 47 | | PM AND PS | | {(D_1_,1), (D_2_,0), (D_3_,0), (D_4_,0), (D_5_,0)} | |
| 48 | | PM AND PM | | {(D_1_,1), (D_2_,0), (D_3_,0), (D_4_,0), (D_5_,0)} | |
| 49 | | PM AND PL | | {(D_1_,1), (D_2_,0), (D_3_,0), (D_4_,0), (D_5_,0)} | |
| 50 | | PL AND NL | | {(D_1_,0.9), (D_2_,0.1), (D_3_,0), (D_4_,0), (D_5_,0)} | |
| 51 | | PL AND NM | | {(D_1_,0.99), (D_2_,0.01), (D_3_,0), (D_4_,0), (D_5_,0)} | |
| 52 | | PL AND NS | | {(D_1_,1), (D_2_,0), (D_3_,0), (D_4_,0), (D_5_,0)} | |
| 53 | | PL AND Z | | {(D_1_,1), (D_2_,0), (D_3_,0), (D_4_,0), (D_5_,0)} | |
| 54 | | PL AND PS | | {(D_1_,1), (D_2_,0), (D_3_,0), (D_4_,0), (D_5_,0)} | |
| 55 | | PL AND PM | | {(D_1_,1), (D_2_,0), (D_3_,0), (D_4_,0), (D_5_,0)} | |
| 56 | | PL AND PL | | {(D_1_,1), (D_2_,0), (D_3_,0), (D_4_,0), (D_5_,0)} | |

**Table 1.** The initial belief rules provided by the expert.

| **Rule number** | | **Rule weight** | ***FlowDiff* and *PressureDiff*** | ***LeakSize* distribution $\{D_{1},D_{2},D_{3},D_{4},D_{5}\}=\{0,2,4,6,8\}$** |  |  |
| --- | --- | --- | --- | --- | --- | --- |
| 1 | | 0.8527 | NL AND NL | {(D_1_,0), (D_2_,0.0515), (D_3_,0.2153), (D_4_,0.2461), (D_5_,0.4871)} |  |  |
| 2 | | 0.0251 | NL AND NM | {(D_1_,0.12), (D_2_,0.2971), (D_3_,0.0445), (D_4_,0.4351), (D_5_,0.1033)} |  |  |
| 3 | | 0.3544 | NL AND NS | {(D_1_,0.0217), (D_2_,0.3724), (D_3_,0.1161),(D_4_,0.2886), (D_5_,0.2012)} |  |  |
| 4 | | 0.1459 | NL AND Z | {(D_1_,0.0823), (D_2_,0.0506), (D_3_,0.1871), (D_4_,0.3646), (D_5_,0.3154)} |  |  |
| 5 | | 0.8451 | NL AND PS | {(D_1_,0.0185), (D_2_,0.0278), (D_3_,0.2202), (D_4_,0.3323), (D_5_,0.4012)} |  |  |
| 6 | | 0.8907 | NL AND PM | {(D_1_,0.0279), (D_2_,0.0339), (D_3_,0.0568), (D_4_,0.4184), (D_5_,0.4630)} |  |  |
| 7 | | 0.8123 | NL AND PL | {(D_1_,0.0057), (D_2_,0.045), (D_3_,0.0235), (D_4_,0.4088), (D_5_,0.517)} |  |  |
| 8 | | 0.2186 | NM AND NL | {(D_1_,0.0753), (D_2_,0.0973), (D_3_,0.0694), (D_4_,0.451), (D_5_,0.307)} |  |  |
| 9 | | 0.2778 | NM AND NM | {(D_1_,0.0671), (D_2_,0.0564), (D_3_,0.09), (D_4_,0.356), (D_5_,0.4305)} |  |  |
| 10 | | 0.4182 | NM AND NS | {(D_1_,0.0309), (D_2_,0.1713), (D_3_,0.0317), (D_4_,0.1322), (D_5_,0.6339)} |  |  |
| 11 | | 0.8033 | NM AND Z | {(D_1_,0.0586), (D_2_,0.1194), (D_3_,0.1059), (D_4_,0.137), (D_5_,0.5791)} |  |  |
| 12 | | 0.0674 | NM AND PS | {(D_1_,0.243), (D_2_,0.4804), (D_3_,0.1474), (D_4_,0.0396), (D_5_,0.0896)} |  |  |
| 13 | | 0.8057 | NM AND PM | {(D_1_,0.1541), (D_2_,0.1287), (D_3_,0.0135), (D_4_,0.113), (D_5_,0.5907)} |  |  |
| 14 | | 0.3853 | NM AND PL | {(D_1_,0.0523), (D_2_,0.1747), (D_3_,0.17), (D_4_,0.2688), (D_5_,0.3342)} |  |  |
| 15 | | 0.7889 | NS AND NL | {(D_1_,0.323), (D_2_,0.0311), (D_3_,0.0222), (D_4_,0.0805), (D_5_,0.5432)} |  |  |
| 16 | | 0.5675 | NS AND NM | {(D_1_,0.0814), (D_2_,0.0846), (D_3_,0.2133), (D_4_,0.3808), (D_5_,0.2399)} |  |  |
| 17 | | 0.1428 | NS AND NS | {(D_1_,0.0876), (D_2_,0.1526), (D_3_,0.5162), (D_4_,0.1305), (D_5_,0.1131)} |  |  |
| 18 | | 0.8843 | NS AND Z | {(D_1_,0.0233), (D_2_,0.0761), (D_3_,0.0212), (D_4_,0.8413), (D_5_,0.0381)} |  |  |
| 19 | | 0.1404 | NS AND PS | {(D_1_,0.1485), (D_2_,0.3432), (D_3_,0.3249), (D_4_,0.0872), (D_5_,0.0962)} |  |  |
| 20 | | 0.7468 | NS AND PM | {(D_1_,0.0415), (D_2_,0.1699), (D_3_,0.2191), (D_4_,0.1227), (D_5_,0.4468)} |  |  |
| 21 | | 0.0345 | NS AND PL | {(D_1_,0.4041), (D_2_,0.137), (D_3_,0.0459), (D_4_,0.1979), (D_5_,0.2151)} |  |  |
| 22 | | 0.1694 | NVS AND NL | {(D_1_,0.0753), (D_2_,0.6399), (D_3_,0.1068), (D_4_,0.0334), (D_5_,0.1446)} |  |  |
| 23 | | 0.0487 | NVS AND NM | {(D_1_,0.1782), (D_2_,0.2108), (D_3_,0.1777), (D_4_,0.0467), (D_5_,0.3866)} |  |  |
| 24 | | 0.6215 | NVS AND NS | {(D_1_,0.6715), (D_2_,0.1594), (D_3_,0.1362), (D_4_,0.0047), (D_5_,0.0282)} |  |  |
| 25 | | 0.6277 | | NVS AND Z | {(D_1_,0.6264), (D_2_,0.2438), (D_3_,0.0082), (D_4_,0.0632), (D_5_,0.0584)} | |
| 26 | | 0.102 | | NVS AND PS | {(D_1_,0.3742), (D_2_,0.421), (D_3_,0.1667), (D_4_,0.0259), (D_5_,0.0122)} | |
| 27 | | 0.3216 | | NVS AND PM | {(D_1_,0.5212), (D_2_,0.2569), (D_3_,0.1259), (D_4_,0.0551), (D_5_,0.0409)} | |
| 28 | | 0.3982 | | NVS AND PL | {(D_1_,0.3265), (D_2_,0.3739), (D_3_,0.1539), (D_4_,0.1261), (D_5_,0.0196)} | |
| 29 | | 0.786 | | Z AND NL | {(D_1_,0.9608), (D_2_,0.0237), (D_3_,0.0079), (D_4_,0.0076), (D_5_,0)} | |
| 30 | | 0.9898 | | Z AND NM | {(D_1_,0.9919), (D_2_,0.0011), (D_3_,0.003), (D_4_,0), (D_5_,0.004)} | |
| 31 | | 0.2544 | | Z AND NS | {(D_1_,0.8551), (D_2_,0.1059), (D_3_,0.0132), (D_4_,0.0208), (D_5_,0.005)} | |
| 32 | | 0.8749 | | Z AND Z | {(D_1_,0.9909), (D_2_,0.0034), (D_3_,0.0035), (D_4_,0.0011), (D_5_,0.0011)} | |
| 33 | | 0.7774 | | Z AND PS | {(D_1_,0.9622), (D_2_,0.017), (D_3_,0.0183), (D_4_,0), (D_5_,0.0025)} | |
| 34 | | 0.7318 | | Z AND PM | {(D_1_,0.9769), (D_2_,0.0082), (D_3_,0.0119), (D_4_,0.0004), (D_5_,0.0026)} | |
| 35 | | 0.4397 | | Z AND PL | {(D_1_,0.9589), (D_2_,0.0196), (D_3_,0.0122), (D_4_,0.0032), (D_5_,0.0061)} | |
| 36 | | 0.3458 | | PS AND NL | {(D_1_,0.2028), (D_2_,0.558), (D_3_,0.0713), (D_4_,0.0671), (D_5_,0.1008)} | |
| 37 | | 0.3049 | | PS AND NM | {(D_1_,0.8126), (D_2_,0.0405), (D_3_,0.0799), (D_4_,0.0524), (D_5_,0.0146)} | |
| 38 | | 0.0119 | | PS AND NS | {(D_1_,0.2447), (D_2_,0.2839), (D_3_,0.1437), (D_4_,0.1717), (D_5_,0.156)} | |
| 39 | | 0.0152 | | PS AND Z | {(D_1_,0.0758), (D_2_,0.3608), (D_3_,0.2793), (D_4_,0.2168), (D_5_,0.0673)} | |
| 40 | | 0.3555 | | PS AND PS | {(D_1_,0.5377), (D_2_,0.1787), (D_3_,0.111), (D_4_,0.1228), (D_5_,0.0498)} | |
| 41 | | 0.2348 | | PS AND PM | {(D_1_,0.3427), (D_2_,0.2938), (D_3_,0.3321), (D_4_,0.02), (D_5_,0.0114)} | |
| 42 | | 0.5284 | | PS AND PL | {(D_1_,0.1897), (D_2_,0.1706), (D_3_,0.3269), (D_4_,0.034), (D_5_,0.2788)} | |
| 43 | | 0.506 | | PM AND NL | {(D_1_,0.0832), (D_2_,0.3687), (D_3_,0.0939), (D_4_,0.3029), (D_5_,0.1513)} | |
| 44 | | 0.8305 | | PM AND NM | {(D_1_,0.0235), (D_2_,0.4773), (D_3_,0.229), (D_4_,0.1995), (D_5_,0.0707)} | |
| 45 | | 0.0565 | | PM AND NS | {(D_1_,0.0117), (D_2_,0.3496), (D_3_,0.2754), (D_4_,0.186), (D_5_,0.1773)} | |
| 46 | | 0.7078 | | PM AND Z | {(D_1_,0.0557), (D_2_,0.309), (D_3_,0.1494), (D_4_,0.2058), (D_5_,0.2801)} | |
| 47 | | 0.4809 | | PM AND PS | {(D_1_,0.1508), (D_2_,0.3133), (D_3_,0.0204), (D_4_,0.2715), (D_5_,0.244)} | |
| 48 | | 0.5969 | | PM AND PM | {(D_1_,0.0341), (D_2_,0.0732), (D_3_,0.2864), (D_4_,0.3405), (D_5_,0.2658)} | |
| 49 | | 0.5852 | | PM AND PL | {(D_1_,0.024), (D_2_,0.052), (D_3_,0.4315), (D_4_,0.1141), (D_5_,0.3784)} | |
| 50 | | 0.7858 | | PL AND NL | {(D_1_,0.0576), (D_2_,0.0451), (D_3_,0.5094), (D_4_,0.3648), (D_5_,0.0231)} | |
| 51 | | 0.6232 | | PL AND NM | {(D_1_,0.357), (D_2_,0.1177), (D_3_,0.0935), (D_4_,0.0301), (D_5_,0.4017)} | |
| 52 | | 0.2792 | | PL AND NS | {(D_1_,0.2499), (D_2_,0.0886), (D_3_,0.4401), (D_4_,0.1973), (D_5_,0.0241)} | |
| 53 | | 0.717 | | PL AND Z | {(D_1_,0.2862), (D_2_,0.2188), (D_3_,0.3114), (D_4_,0.072), (D_5_,0.1116)} | |
| 54 | | 0.063 | | PL AND PS | {(D_1_,0.5321), (D_2_,0.0826), (D_3_,0.1025), (D_4_,0.1316), (D_5_,0.1512)} | |
| 55 | | 0.5924 | | PL AND PM | {(D_1_,0.0457), (D_2_,0.1474), (D_3_,0.2638), (D_4_,0.1933), (D_5_,0.3498)} | |
| 56 | | 0.4286 | | PL AND PL | {(D_1_,0.0669), (D_2_,0.3833), (D_3_,0.2152), (D_4_,0.2442), (D_5_,0.0904)} | |

**Table 2.** The belief rules after optimization.
